# *Plasmodium berghei* P47 is essential for ookinete protection from the *Anopheles gambiae* complement-like response

**DOI:** 10.1038/s41598-017-05917-6

**Published:** 2017-07-20

**Authors:** Chiamaka Valerie Ukegbu, Maria Giorgalli, Hassan Yassine, Jose Luis Ramirez, Chrysanthi Taxiarchi, Carolina Barillas-Mury, George K. Christophides, Dina Vlachou

**Affiliations:** 1 Vector Immunogenomics and Infection Laboratory, Department of Life Sciences, Imperial College London, London, United Kingdom; 2 Laboratory of Malaria and Vector Research, National Institute of Allergy and Infectious Diseases, National Institutes of Health, Rockville, Maryland United States of America; 3 National Center for Agricultural Utilization Research, Crop Bio-protection Research Unit, Agricultural Research Service, United States Department of Agriculture, Peoria, Illinois United States of America

## Abstract

Malaria is a mosquito-borne disease affecting millions of people every year. The rodent parasite *Plasmodium berghei* has served as a model for human malaria transmission studies and played a pivotal role in dissecting the mosquito immune response against infection. The 6-cysteine protein P47, known to be important for *P. berghei* female gamete fertility, is shown to serve a different function in *Plasmodium falciparum*, protecting ookinetes from the mosquito immune response. Here, we investigate the function of *P. berghei* P47 in *Anopheles gambiae* mosquito infections. We show that P47 is expressed on the surface of both female gametocytes and ookinetes where it serves distinct functions in promoting gametocyte-to-ookinete development and protecting ookinetes from the mosquito complement-like response, respectively. The latter function is essential, as ookinetes lacking P47 are targeted for killing while traversing the mosquito midgut cells and eliminated upon exposure to hemolymph proteins of the complement-like system. Silencing key factors of the complement-like system restores oocyst development and disease transmission to rodent hosts. Our data establish a dual role of *P. berghei* P47 *in vivo* and reinforce the use of this parasite to study the impact of the mosquito immune response on human malaria transmission.

## Introduction

Malaria remains a great global health problem affecting millions of people and killing over 400,000 every year. The most malignant of human malaria parasites is *P. falciparum* that causes complicated and cerebral malaria affecting mostly children age 2–10 and pregnant women. In sub-Saharan Africa, where the vast majority of malaria mortalities, morbidities and financial burden are recorded, the main vector of *P. falciparum* is the mosquito *A. gambiae*.

The rodent malaria parasite *P. berghei* has served for decades as a model for human malaria transmission owing to its significant genomic synteny^1, 2^ and extensive gene orthology^3^ with *P. falciparum*. This is despite the functional non-equivalence of processes relating to immunopathology and virulence of asexual parasite stages in the vertebrate host. In particular, *P. berghei* infects well mosquito vectors of human malaria, including *A. gambiae*, allowing the study of the full transmission cycle through rodent hosts, and it is genetically tractable and biologically safe. As a result, *P. berghei* has been fundamental in dissecting the *A. gambiae* immune system and delineating its importance in malaria transmission^4^. Nevertheless, concerns have been raised as to whether some of the data obtained with *P. berghei* infections can be directly relevant to human malaria transmission, owing to differences in how certain modules of the *A. gambiae* immune system deal with *P. berghei* and *P. falciparum* infections^5–9^. Such differences are thought to be primarily shaped by geographic and co-evolutionary adaptation between vectors and parasites^10–15^. Indeed, the natural vector of *P. berghei* is believed to be *Anopheles dureni* that is found only in Central African highland forests. Furthermore, experimental mosquito infections with *P. berghei* are performed at temperatures that are 6–7 °C lower than those used for *P. falciparum* infections. This temperature difference is shown to significantly impact on the mosquito physiology and affect certain immune reactions^16^, potentially accounting for some of the observed differences in the mosquito response against the two parasites.

*Plasmodium* expresses a variety of plasma membrane or surface proteins that play key roles in interactions with host cells or between parasite cells, promoting infection, replication and transmission^4, 17^. Amongst them are members the s48/45 domain 6-cysteine (6-cys) protein family that are expressed in stage-specific fashions^18, 19^. The gametocyte-expressed 6-cys proteins P47, P48/45 and P230 are shown to play important roles in fertilization that takes place inside the mosquito midgut lumen leading to ookinete development^20, 21^. In particular, P47 is important for *P. berghei* female gamete fertility, a function shown to be essential for fertilization in *in vitro* parasite cultures^21^. Paradoxically, this function appears not to be shared by *P. falciparum* P47 (Pfs47) that is dispensable for fertilization^22^. Instead, Pfs47 is shown to mediate suppression of c-Jun N-terminal kinase (JNK) signaling in *A. gambiae* invaded midgut cells, inhibiting ookinete nitration and subsequent elimination by reactions of a complement-like pathway mediated by the C3-like protein TEP1^23, 24^. Here, we set to elucidate the function of *P. berghei* P47 (PbP47) *in vivo* and assess the relevance of this parasite in studying the role of the mosquito immune response in human malaria transmission. We demonstrate that PbP47 has a dual function in early stages of *A. gambiae* infection, promoting gametocyte-to-ookinete and ookinete-to-oocyst development, respectively. The latter function is essential and protects ookinetes from the mosquito complement-like response.

## Results

### PbP47 expression in female gametocytes and ookinetes

**Figure 1:**
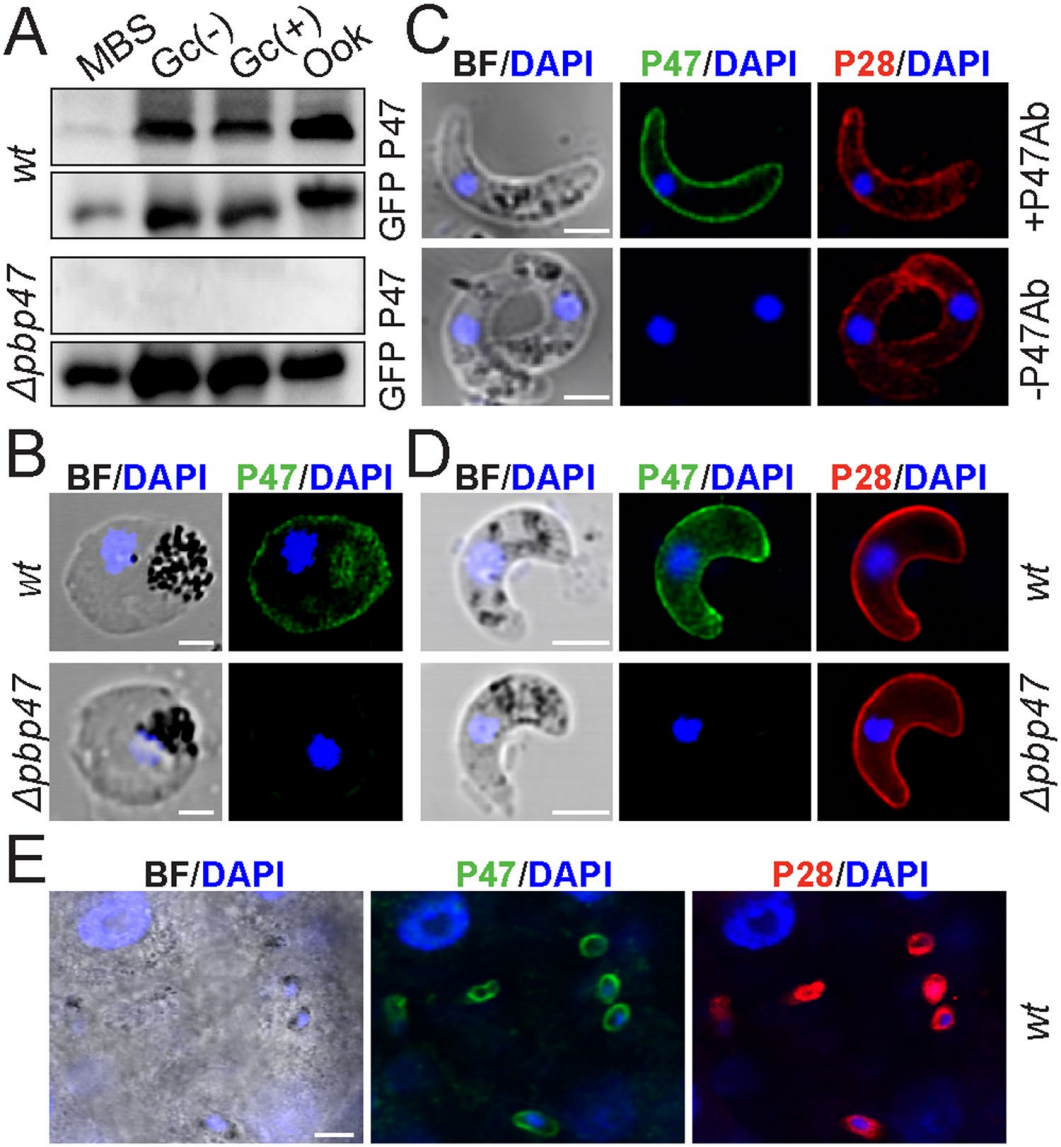
Expression analysis of *P. berghei* P47. (**A**) Western blot analysis of protein extracts of *ANKA 507m6cl1* (*wt*) and *Δpbp47* parasites using the PbP47 and GFP (control) antibodies. A cropped picture of the blot is presented; the full-length blot is shown in Supplementary Fig. S1. MBS, purified mixed blood stages; Gc(−), non-activated gametocytes; Gc(+), activated gametocytes; Ook, *in vitro* produced ookinetes 24 hours post activation. (**B–E**) Immunofluorescence assays from confocal sections of *ANKA 507m6cl1* (*wt*) non-activated gametocytes (**B**), *in vitro* produced ookinetes 24 hours post activation (**C**), ookinetes in the blood bolus of *A. gambiae* mosquitoes 20–22 hours post blood feeding (**D**), and ookinetes traversing the mosquito gut epithelium 24 hours post blood feeding (**E**), stained with the PbP47 antibody (green), DAPI (blue) and P28 antibody (red). BF is bright field. The *Δpbp47* parasite line is used as a negative control for gametocyte and blood bolus ookinete imaging, and non-primary antibody staining was used as a control for *in vitro* produced ookinetes (−P47Ab). Images were taken at 630x magnification for (**B–D**) and 400x magnification for (**E**). Scale bar is 2.5 µm in (**B,C**), 5 µm in (**D**) and 10 µm in (**E**).

We generated a rabbit polyclonal antibody against the PbP47 coding region lacking the signal peptide and the C-terminal hydrophobic domain (amino acids 30–412) and used it in western blot and immunofluorescence assays. In these assays, we used the *ANKA 507m6cl1 P. berghei* line that constitutively expresses GFP but is otherwise wild type (*wt)* ^25^ and the *Δpbp47* line that lacks *PbP47* and also expresses GFP throughout the parasite life cycle^21^. The results showed that PbP47 was detected in almost equal abundance in total protein extracts from both non-activated and activated female gametocytes and *in vitro* cultured ookinetes of the *wt* line but was absent from gametocytes and ookinetes of the *Δpbp47* line (Fig. 1A and Supplementary Fig. S1). In immunofluorescence assays, PbP47 was detected on the surface of female gametocytes (Fig. 1B) and ookinetes, both *in vitro* (Fig. 1C) and in the blood bolus of *A. gambiae* mosquitoes that had been fed on infected mice 20–22 hours earlier (Fig. 1D), exhibiting a distribution similar to the P28 ookinete surface protein. A similar surface distribution was detected in ookinetes while traversing the mosquito midgut epithelium (Fig. 1E).

### PbP47 is essential for ookinete protection from mosquito complement-like reactions

We examined the infectivity of *Δpbp47* parasites in *A. gambiae* mosquitoes of the N’gousso strain by enumerating oocysts developing in the mosquito midguts 10 days post infection. In the same set of experiments, the mosquito complement-like system was silenced through injections of double stranded RNA (dsRNA) of genes encoding two proteins of the mosquito complement-like pathway, TEP1^26^ and LRIM1^27^. A disulphide-bonded complex of LRIM1 and its structural and functional homolog APL1C are shown to interact with the mature form of TEP1 (TEP1_cut_) preventing its reaction with the hemolymph or self-tissues and directing it to the ookinete surface mediating their lysis or melanization^28, 29^. For each of the *TEP1* and *LRIM1* set of dsRNA injections, control mosquitoes were injected with equal amounts of dsRNA of the *Escherichia coli LacZ* gene.

**Figure 2:**
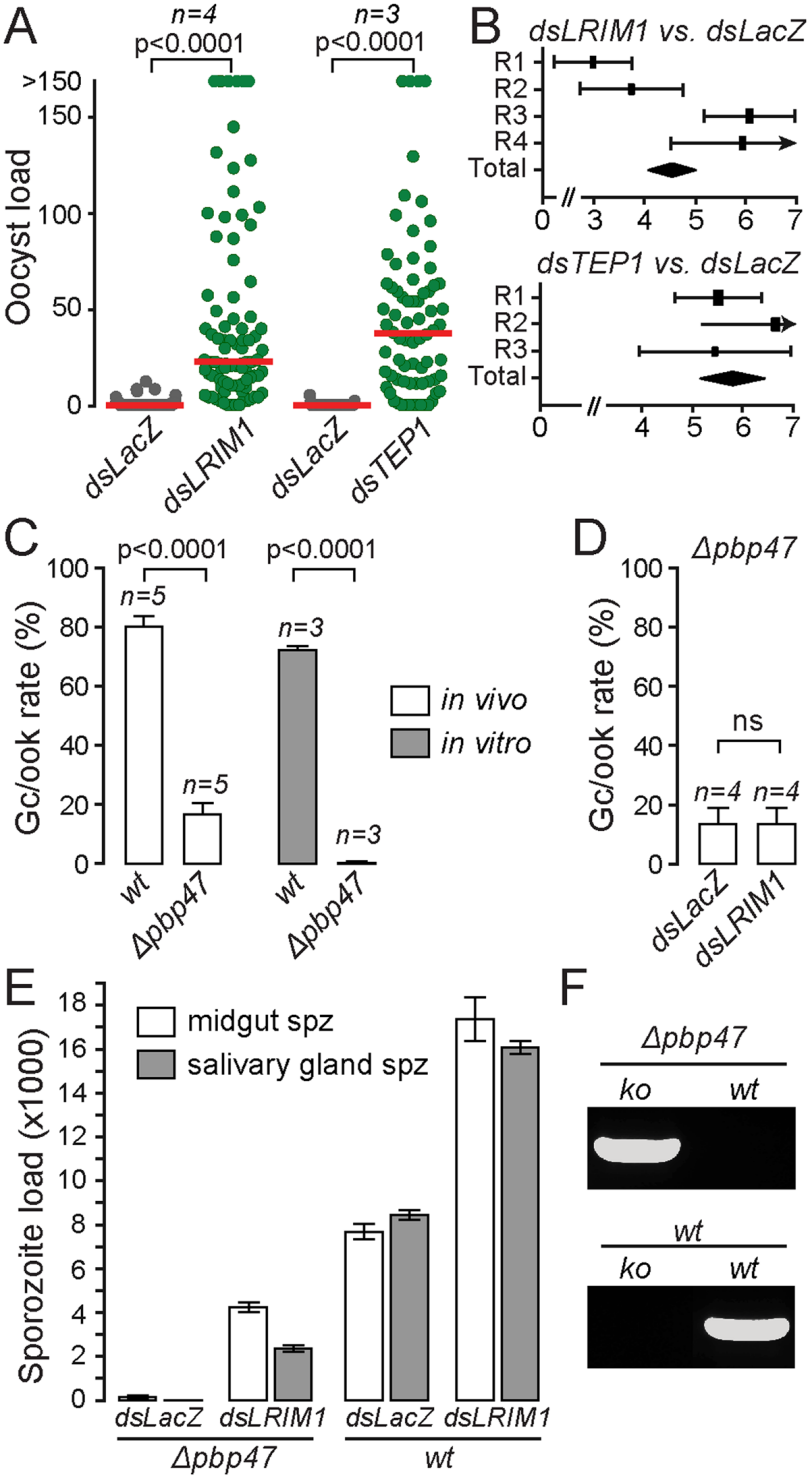
Phenotypic analysis of the *Δpbp47* parasite line in *A. gambiae* infections. (**A**) Effect of *dsLacZ* injections (control) and *LRIM1* and *TEP1* silencing on *Δpbp47* oocyst. The median number of oocysts is shown with a red line. Statistical significance was determined using the Mann-Whitney *U*-test. (**B**) Forest plots of GLMM analyses of the infections shown in *A*. The variation of the fixed effect estimate in each (squares) and all (diamonds) replicates (R) is shown ( ± 95% confidence interval, glmmADMB). The square size is proportional to the sum of midguts analyzed in each replicate. (**C**) *In vivo* and *in vitro* gametocyte-to-ookinete (Gc/ook) conversion rates of *Δpbp47* and control *ANKA 507m6cl1* (*wt*). (**D**) *In vivo* gametocyte-to-ookinete (Gc/ook) conversion rates of *Δpbp47* parasites in *dsLacZ* (control) and *dsLRIM1* injected mosquitoes. In both (**C**) and (**D**), the number of replicates (n) and standard deviation are shown. Statistical significance was determined with a two tailed, unpaired Student’s *t*-test (ns, non-significant). (**E**) Midgut and salivary gland *Δpbp47* and *ANKA 507m6cl1* (*wt*) sporozoite numbers in control *dsLacZ* and *dsLRIM1* injected *A. gambiae*. The mean of the pooled data from two biological replicates is presented. For each replicate, sporozoite numbers were determined from 25 midguts or salivary glands at days 15 and 21 post blood feeding, respectively. Error bars show standard error. (**F**) PCR genotypic analysis of the *Δpbp47* knockout (*ko*) or *wt* locus on blood stage parasites of the *Δpbp47* and *ANKA 507m6cl1* (*wt*) parasite lines following transmission from *LRIM1* silenced *A. gambiae*.

The results showed that the mean/median intensities of infection with the *Δpbp47* line in the two sets of *dsLacZ*-injected mosquitoes were 0.52/0 and 0.14/0, respectively (Fig. 2A,B and Supplementary Table S1). Only 16% and 8% of the midguts assessed harbored any oocysts. Similar results were obtained in mosquitoes not injected with dsRNA (Supplementary Fig. S2), confirming that this is not an effect of injury that is known to activate mosquito immunity^30^. However, when *LRIM1* or *TEP1* were silenced, a large number of morphologically fully developed *Δpbp47* oocysts were observed, with mean/median intensities of 49.5/22 and 46.6/36.5, respectively (Fig. 2A,B and Supplementary Table S1). The infection prevalence also increased from 16% and 8% in *dsLacZ*-injected controls to 96% in *LRIM1* and 89% in *TEP1* knockdown mosquitoes, respectively. Parallel infections with the parental *wt P. berghei* line *ANKA 507m6cl1* were also carried out as a control to the gene silencing experiments (Supplementary Fig. S3 and Supplementary Table S1). The results showed mean/median infection intensities of 238.1/200 (92% infection prevalence) in *LRIM1* and 397.5/397 (100% infection prevalence) in *TEP1* silencing. In comparison, *dsLacZ*-injected controls showed mean/median infection intensities of 23.7/13 (87% infection prevalence) and 33.7/26 (85% infection prevalence), respectively.

As an additional control for the function of *P. berghei* P47 in parasite protection from the mosquito complement-like response, we also performed *A. gambiae* infections with a *P. berghei* line that lacks the gene encoding the P48/45 protein. Like P47, P48/45 belongs to the 6-cysteine family of proteins and is shown to be important for male gamete fertility^20, 21^. The data showed that the increase of *Δpbp48/45* infection intensities in *TEP1* kd compared to *dsLacZ* injected control mosquitoes was very small, as the median oocyst number in two independent biological replicates was 2 and 1, respectively (Supplementary Table S2).

The smaller number of *Δpbp47* compared to *ANKA 507m6cl1* oocysts in *TEP1* and *LRIM1* knockdown mosquitoes pointed to an additional, non-essential, function of PbP47 prior to mosquito midgut invasion, presumably in fertilization as shown previously^21^. To investigate this, we measured the gametocyte-to-ookinete conversion rate of *Δpbp47* parasites *in vivo* in the *A. gambiae* midgut bolus at 20–22 hours post mosquito blood feeding. Mosquito infections with the *ANKA 507m6cl1 P. berghei* line served as *wt* control. A significant 4.2-fold difference in gametocyte-to-ookinete rate was observed between the two parasites, ranging from 72% to 83% (average 80%) in *wt* and 16% to 20% (average 17%) in *Δpbp47* parasites in a total of five biological replicates (Fig. 2C). Nevertheless, the *in vivo* gametocyte-to-ookinete rate of *Δpbp47* parasites was substantially higher than the one observed *in vitro* that was virtually abolished ranging from 0% to 0.4% (average 0.1% from 3 biological replicates).

We investigated whether the reduced gametocyte-to-ookinete conversion rate of *Δpbp47* parasites is also affected by reactions of the mosquito complement-like system. This hypothesis would require complement-mediated parasite killing to occur in the midgut lumen, which is against the general understanding that the complement-like pathway functions only in the hemolymph. Indeed, *LRIM1* silencing could not restore the *Δpbp47* gametocyte-to-ookinete conversion rate (15.3% ± 5.3% from 4 biological replicates) that remained similar to that recorded in control *dsLacZ* injected mosquitoes (15.4% ± 5.6% from 4 biological replicates; Fig. 2D). These data falsified this hypothesis and revealed a dual function of PbP47 in ookinete development in the mosquito midgut lumen and ookinete protection from mosquito complement reactions. Whilst the former function is non-essential *in vivo*, the latter function is essential and its disruption abolishes transmission.

### Silencing the mosquito complement-like pathway restores transmission of *Δpbp47* parasites

We examined whether the *Δpbp47* oocysts developing upon silencing of the mosquito complement-like system can produce sporozoites that subsequently infect the vertebrate host and complete their transmission cycle. The numbers of sporozoites in pools of 25 midguts and salivary glands were assessed 15 and 21 days post infection, respectively, in two independent biological replicates. A very small number of *Δpbp47* sporozoites (42 ± 29 per midgut) were observed in the midguts of *dsLacZ*-injected control mosquitoes, compared to the typically high number of sporozoites (7,790 ± 785) of the *ANKA 507m6cl1* line (Fig. 2E and Supplementary Table S3). No *Δpbp47* sporozoites were observed in the salivary glands of *dsLacZ-*injected mosquitoes. However, approximately 100-fold increase in *Δpbp47* sporozoite numbers (4,156 ± 464) was recorded in the midguts of *LRIM1* knockdown mosquitoes 15 days post infection compared to the number of sporozoites in midguts of *dsLacZ*-treated mosquitoes. About half of these sporozoites (2,398 ± 79) reached the salivary glands at day 21 post infection. These numbers were lower than those of *ANKA 507m6cl1* sporozoites in the midguts (17,343 ± 1,988) and salivary glands (16,060 ± 665) of *LRIM1* knockdown mosquitoes, reflecting the observed differences in oocyst loads that are due to the early function of PbP47 in fertilization.

The infectivity of *Δpbp47* sporozoites in *LRIM1* knockdown mosquitoes was assessed by allowing mosquitoes 21 days post blood feeding to feed on C57/BL6 mice in bite-back experiments (mosquito-to-mouse transmission) and monitoring the parasitaemia in mice for 14 consecutive days post mosquito bite. Like *ANKA 507m6cl1* (*wt*) sporozoites, *Δpbp47* sporozoites could be readily transmitted to all 6 mice producing asexual blood stage parasites (Table S2). PCR analysis on genomic DNA extracted from blood stage parasites from these mice confirmed that parasites encompassed the *Δpbp47* locus and that there was no contamination with the *wt* locus that was absent from all the mice (Fig. 2F). None of the 6 mice used was infected after bite-back experiments using *dsLacZ*-injected mosquitoes infected with *Δpbp47* parasites, consistent with the data showing that *Δpbp47* parasites could not reach the mosquito salivary glands.

### *Δpbp47* ookinetes are eliminated upon midgut traversal and binding of TEP1

**Figure 3:**
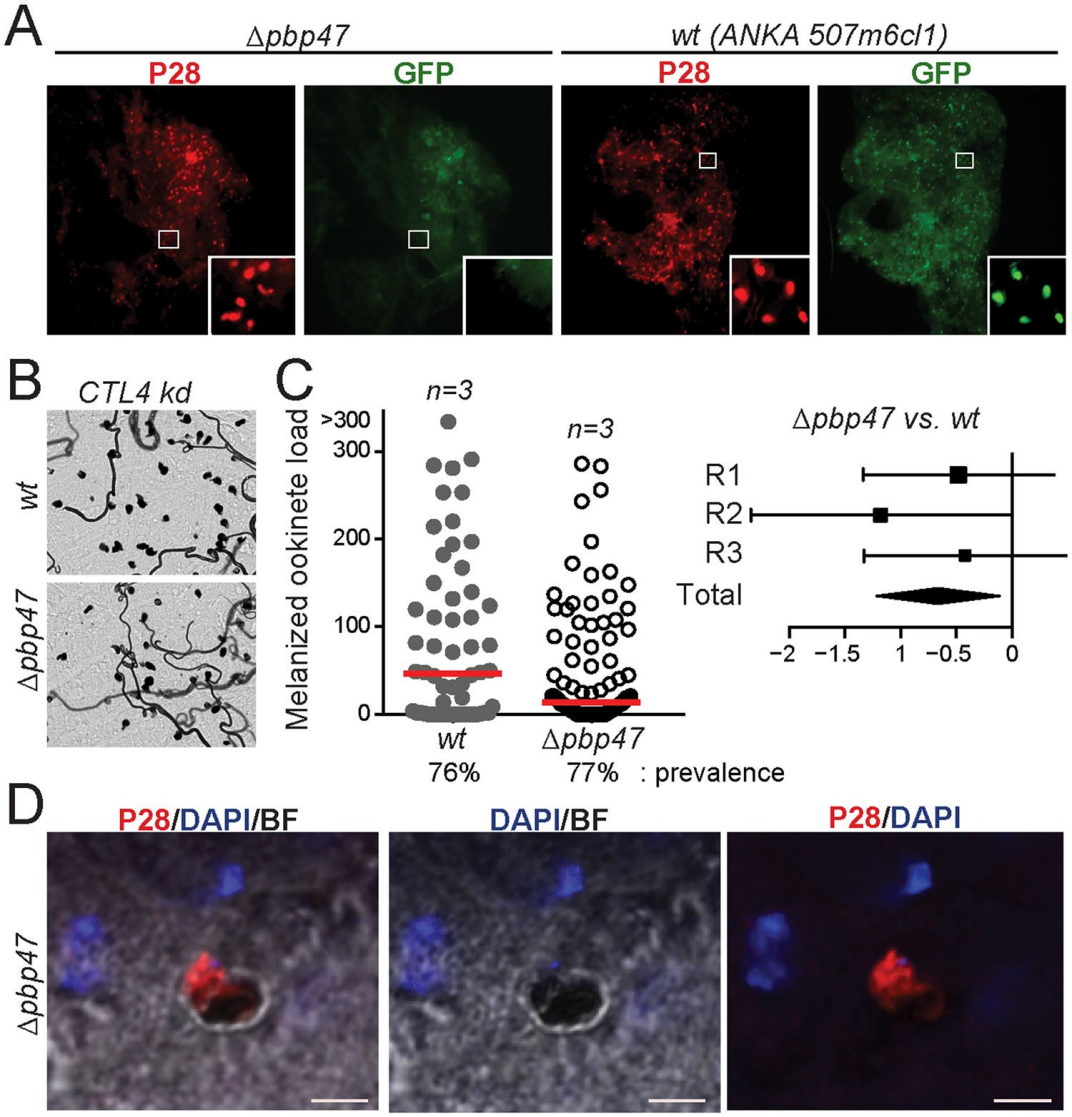
Midgut invasion assays of *Δpbp47* parasites. (**A**) Fluorescence microscopy of *ANKA 507m6cl1* (*wt*) and *Δpbp47* ookinetes stained with P28 antibody in the *A. gambiae* midgut epithelium 24–26 hours post blood feeding. Images are taken at 100x magnification, and inset images at 400x magnification. (**B**) Light microscopy images (100x magnification) of melanized *ANKA 507m6cl1* (*wt*) and *Δpbp47* parasites in *CTL4* silenced *A. gambiae* mosquito midguts. (**C**) Left: load of melanized *ANKA 507m6cl1* (*wt*) and *Δpbp47* ookinetes in the midguts of *CTL4* silenced mosquitoes (left). Red lines show the median ookinete load. Right: forest plots of melanized ookinete loads as determined by GLMM analysis. The variation of the fixed effect estimate in each (squares) and all (diamonds) replicates (R) is shown ( ± 95% confidence interval, glmmADMB). The square size is proportional to the sum of midguts analyzed in each replicate. (**D**) Confocal images (630x magnification) of a *Δpbp47* melanized ookinete in the midgut of *CTL4* silenced *A. gambiae*, stained with the P28 antibody (red) and DAPI (blue). BF is bright field. Scale bar is 5 µm.

We carried out fluorescence microscopy of *A. gambiae* midguts infected with *Δpbp47* or control *ANKA 507m6cl1* parasite lines, both expressing GFP throughout their life cycle, to confirm that *Δpbp47* ookinetes developing in the mosquito midgut lumen traverse the midgut epithelium and are killed prior to their transformation to oocysts. It has been previously shown that, during midgut traversal, ookinetes killed by complement-like reactions are rapidly depleted from GFP fluorescence but continue to exhibit P28 on their surface^31^. Infected midguts were dissected at 24–26 hours post blood feeding, a time that coincides with epithelium traversal, and visualized after staining with an antibody against P28. Indeed, a large number of P28-positive but GFP-negative *Δpbp47* ookinetes were detected (Fig. 3A and Supplementary Table S4). These results confirmed that the *Δpbp47* ookinetes invade the mosquito midgut epithelium and are killed upon midgut epithelium traversal.

To investigate whether the *Δpbp47* ookinetes are killed during or immediately after midgut epithelium traversal, we infected *C-type lectin 4* (*CTL4*) knockdown and control *dsLacZ*-injected mosquitoes with *Δpbp47* or control *ANKA 507m6cl1* parasites. CTL4 is a key regulator of ookinete elimination following attack by the complement-like system, promoting parasite lysis as opposed to melanization^27^. Therefore, silencing *CTL4* results in melanization of ookinetes that are otherwise lysed and eliminated. This reaction occurs immediately after ookinetes have traversed the midgut epithelium and can consequently be used to readily visualize the dead ookinetes. The results revealed that the *Δpbp47* ookinetes are indeed melanized as soon as they traverse the midgut epithelial wall (Fig. 3B). The number of melanized *Δpbp47* ookinetes was again significantly lower than the respective number of *wt* control *ANKA 507m6cl1* ookinetes that infected the same batch of *CTL4* knockdown mosquitoes (Fig. 3C and Supplementary Table S5). These data indicate that the fertilization and ookinete killing phenotypes associated with *PbP47* knockout are independent processes occurring in different mosquito compartments, the midgut lumen and the hemolymph-bathed sub-epithelial space, respectively. Indeed, several instances of ookinetes showing melanization of their apical end that is presumably exposed to the hemolymph were captured, with their rear end still exhibiting staining with the surface protein P28 (Fig. 3D).

**Figure 4:**
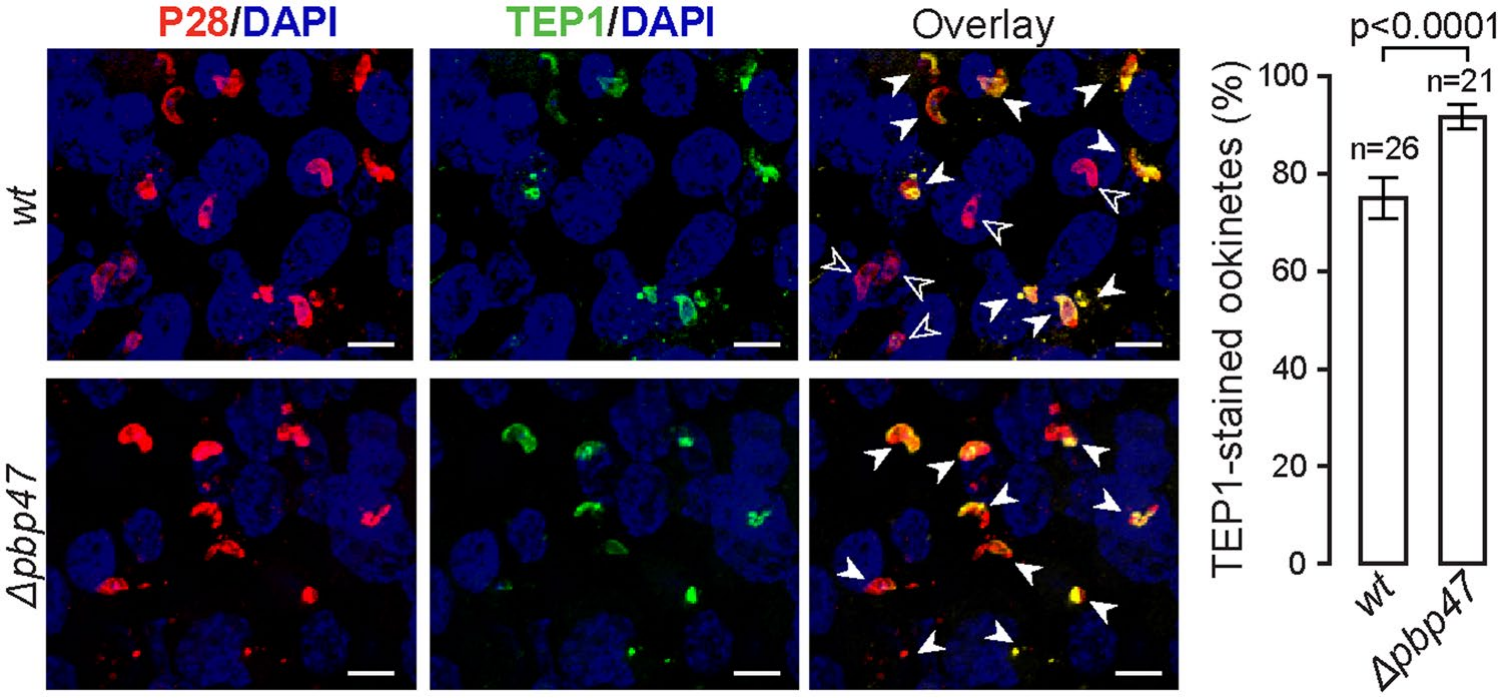
*ANKA 507m6cl1* (*wt*) and *Δpbp47* ookinete killing by complement-like reactions. Tissues are stained with P28 (red) and TEP1 (green) antibodies and DAPI. P28 staining (open arrowhead) marks all ookinetes, while TEP1 and P28 double staining marks ookinetes that are either killed or in the process of being killed. Images were taken at 400x magnification. Scale bar is 10 µM. The percentage of double-stained ookinetes is shown on the graph on the right, where n is the total number of midguts analyzed in three independent biological replicates.

To investigate the mechanism of complement-mediated killing of *Δpbp47* ookinetes, midgut epithelia of naïve mosquitoes infected with *Δpbp47* or *ANKA 507m6cl1* control parasites were dissected at 27–30 hours post blood feeding and stained with antibodies against P28 and TEP1. Whilst P28 is found in all ookinetes, TEP1 marks ookinetes that are either dead or in the process of being killed^31^. We carried out three independent biological replicate infections and analyzed a total of 4,311 (21 midguts) and 19,421 (26 midguts) *Δpbp47* and *ANKA 507m6cl1* ookinetes, respectively (Fig. 4). The results revealed that the fraction of TEP1-stained *Δpbp47* ookinetes (91.8% ± 4.4%) was significantly higher than the fraction of TEP1-stained *ANKA 507m6cl1* ookinetes (77.1% ± 8.4%). These data support the hypothesis that exaggerated TEP1 binding during mosquito midgut traversal is indeed the elimination mechanism of ookinetes lacking P47.

### *Δpbp47* ookinetes are targeted for elimination whilst traversing the midgut cells

**Figure 5:**
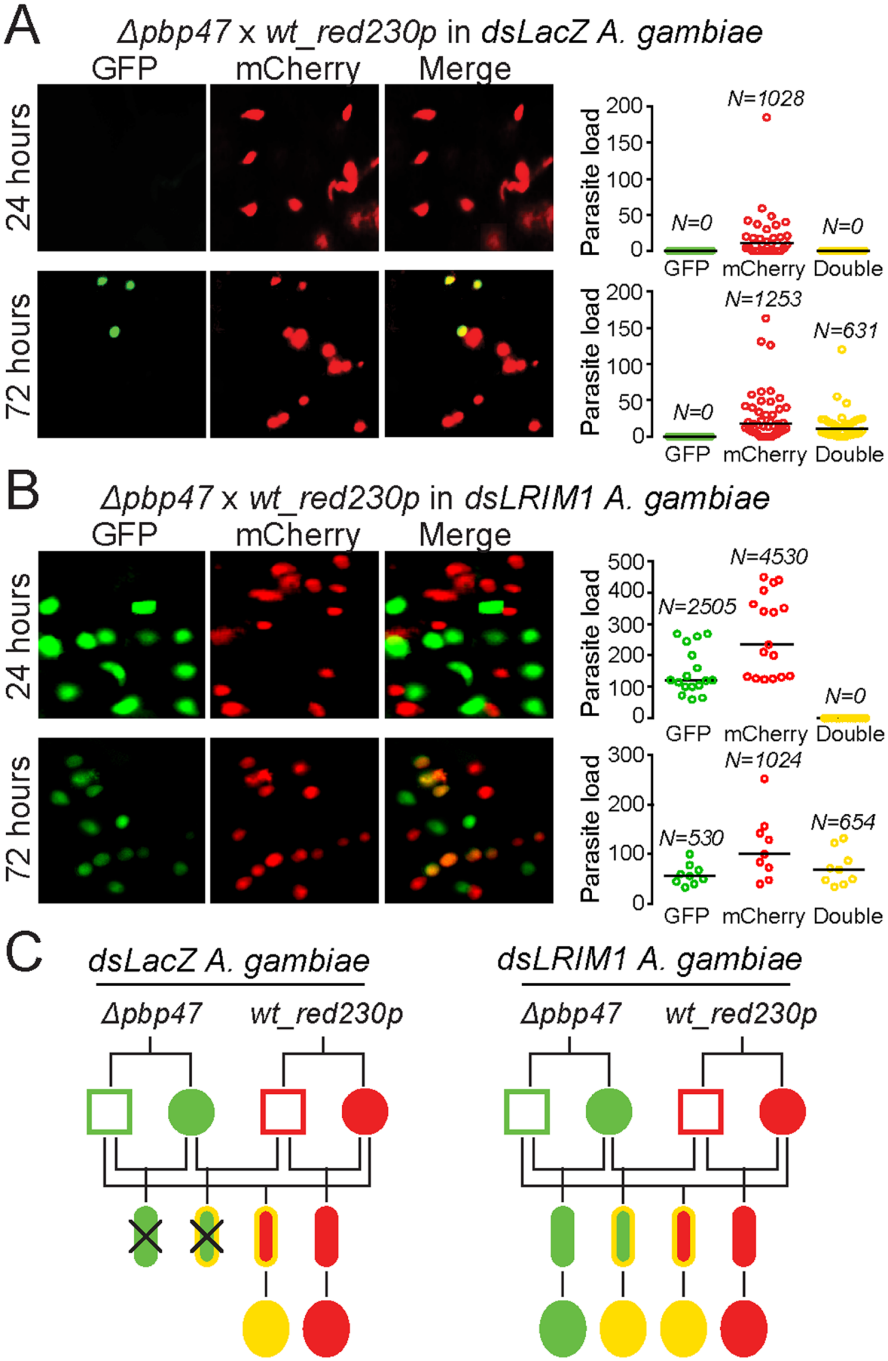
*A. gambiae* co-infections with *Δpbp47* and *wt_red*_*230p*_ parasite lines. Mosquitoes injected with *dsLacZ* (**A**) or *dsLRIM1* (**B**) were fed on mice co-infected with GFP-expressing *Δpbp47* and mCherry-expressing *wt_red*_*230p*_ parasites. Parasites in the mosquito midguts were enumerated at 24 and 72 hours post blood feeding. The left composite panel consists of representative fluorescence microscopy images (GFP, mCherry and combination of the two channels). The graphs on the right show the load of GFP-positive, mCherry-positive and GFP/mCherry double-positive parasites per midgut at each time point. Horizontal black lines indicate the median parasite load. Data from three independent biological replicates are pooled; *N* is the total number of parasites counted. (**C**) Genograms summarizing the results of *A* and *B* in *dsLacZ* (left) and *dsLRIM1* (right) injected mosquitoes. Squares and circles correspond to male and female gametocytes, respectively; small ellipses correspond to ookinetes; large ellipses correspond to early stage oocysts. The outline color indicates the genotype: green is *Δpbp47* homozygotes, red is *wt_red*_*230p*_ homozygotes and yellow is *Δpbp47/wt_red*_*230p*_ heterozygotes. The fill-in color indicates the phenotype: green is GFP-positive, red is mCherry-positive and yellow is GFP/mCherry double-positive parasites. Crossed parasites are killed and not detected.

We investigated the mechanism of P47-mediated protection of ookinetes using an assay whereby the GFP-expressing *Δpbp47* parasite line was used together with the mCherry-expressing *wt_red*_*230p*_ line^32^ in co-infections of control *dsLacZ-*injected and *LRIM1* knockdown mosquitoes. Our testable hypothesis based on the aforementioned model was that the presence of P47 on mCherry-expressing *wt* ookinetes would enable some of the GFP-expressing *Δpbp47* ookinetes to successfully traverse the midgut epithelium and develop to oocysts by suppressing the signaling response that leads to ookinete nitration. Mosquitoes were fed on mice co-infected with the two lines, and fluorescent ookinetes in the midgut epithelium were enumerated at 24 hours post blood feeding (Fig. 5). The results revealed that only mCherry-expressing ookinetes were detected in control *dsLacZ-*injected mosquito midguts. We had previously shown that the male alleles of the constitutively expressed GFP and mCherry transgenes (both under the transcriptional control of the *ef1α* promoter) are silent during the first 32 hours post mosquito blood feeding^32^. Thus ookinete fluorescence is either inherited as maternal mRNA from female gametocytes or newly provided through expression of the female alleles in the zygote/ookinete. Accordingly, the observed mCherry-expressing ookinetes originate from self-fertilization of *wt_red*_*230p*_ gametes and cross-fertilization of *wt_red*_*230p*_ female and *Δpbp47* male gametes; both ookinete genotypes carry functional P47 female alleles.

Double mCherry and GFP expressing oocysts were detected in the mosquito midguts at 72 hours post blood feeding. These oocysts originated from cross-fertilization of *wt_red*_*230p*_ female and *Δpbp47* male gametes, and began expressing the GFP reporter inherited from the male gamete soon after the ookinete transformation to oocyst^32^. When *LRIM1* was silenced, both GFP and mCherry expressing ookinetes were detected in the midgut epithelium 24 hours post blood feeding. Numerous mCherry as well as GFP and double fluorescent oocysts were detected at 72 hours post blood feeding. These data demonstrate that P47-expressing ookinetes cannot rescue co-invading ookinetes lacking P47 and that P47 protects from complement-mediated elimination only when present on the ookinete surface. The results also reveal that attack of ookinetes lacking P47 by the complement-like system does not affect co-invading ookinetes that express P47 on their surface.

## Discussion

Our data establish a dual function of *P. berghei* P47 in early stages of mosquito infection. The first function pertains to the gametocyte-to-ookinete development that takes place in the mosquito midgut lumen, presumably in female gamete fertility as reported previously^21^. This function is essential for *in vitro* ookinete culturing but not *in vivo* in the mosquito midgut; its disruption is nonetheless accountable for a great reduction in ookinete numbers. The second function is during the ookinete-to-oocyst transition, which coincides with midgut cell traversal, and is associated with ookinete protection from the mosquito immune response. This function is essential as P47 disruption results in parasite transmission blockade that can be averted by silencing key factors of the mosquito complement-like system such as TEP1 and LRIM1. A similar function has been previously reported for *P. falciparum* P47^24^, which paradoxically appears to be dispensable for fertilization^22^.

The mosquito complement-like response is shown to be responsible for a great reduction of ookinete numbers. The complement C3-like effector, TEP1, attacks ookinetes as soon as these come into contact with the hemolymph that fills the basal sub-epithelial space and the extensive network of crypts formed by invaginations of the basal plasma membrane known as the basal labyrinth. Whether ookinete attack and elimination is a stochastic or deterministic process is an area of intense research. Whilst wild-type ookinete elimination rates can vary between infections suggesting some level of stochasticity, the discoveries that *P. falciparum* and *P. berghei* P47 promote ookinete resistance to this response support the idea of specific mosquito-parasite interactions that greatly affect the infection outcome. In the model proposed for the function of *P. falciparum* P47, the presence of P47 on the ookinete surface suppresses activation of JNK signaling in the invaded midgut epithelium^24^. JNK signaling is thought to trigger apoptosis of the invaded cells through activation of effector caspases. It also induces the production of heme peroxidase 2 (HPX2) and NADPH oxidase 5 (NOX5), which together potentiate nitration of ookinetes marking them for destruction by reactions of the mosquito complement-like system^33^. Disruption of the JNK pathway in *A. gambiae*, by silencing activators of this cascade, greatly enhances infection^34^. Over-activation of the JNK pathway, by silencing its suppressor Puckered, enhances midgut nitration and greatly reduces the intensity and prevalence of infection, an effect that is reverted by co-silencing HPX2, NOX5 or TEP1.

Our finding that P47 presence on the ookinete surface does not offer immune protection to co-invading ookinetes lacking P47 and, *vice versa*, that P47 absence from the ookinete surface does not affect co-invading ookinetes expressing P47 provide additional insights to this model. To this end, it appears that P47 functions locally by protecting ookinetes from immune targeting inside the invaded midgut cell rather than systemically by suppressing activation of JNK signaling in the midgut epithelium or the complement-like pathway in the hemolymph. It has been recently shown that P47 is not the sole mediator of immune resistance of African *P. falciparum* ^12^, suggesting that the ookinete surface as a whole may have evolved to avert or tolerate immune targeting and elimination. When a major surface protein is missing, the ookinete may be recognized as foreign triggering nitration followed by TEP1 attack.

Together our findings reconcile the role of P47 in *P. berghei* and *P. falciparum* in protecting ookinetes from the complement-like response of the mosquito vector lending additional confidence to the use of *P. berghei* as a model laboratory system to study the impact of the mosquito immune response on malaria transmission.

## Methods

### Ethics statement

All animal experimental procedures were reviewed and approved by the Imperial College London Animal Welfare and Ethical Review Body (AWERB) and the United Kingdom Home Office. These procedures were in accordance with the Animal Scientifics Procedures Act 1986, under the UK Home Office Licenses PLL70/7185 and PPL70/8788.

### Parasite cultivation and infections

Parasite lines included *Δpbp47* (765acl1; RMgm347^21^), also known as *Δp47_green*_*230p*_^32^, *Δpbp48/45* (764acl1; RMgm346), *ANKA 15cy1A* line (507m6cl1)^25^, also known as *wt_green*_*230p*_^32^, and *wt_red*_*230p*_^32^. Parasite culturing and purification were carried out using standard procedures^35^. Mosquito infections of the *A. coluzzii* N’gousso strain (*A. gambiae* M form*)* were carried out by feeding on mice with 5–6% parasitaemia and 1–2% gametocytaemia. Co-infections were carried out by feeding on mice infected with mouse blood samples of equal parasitaemia. For mosquito to mouse transmission 50–60 mosquitoes 21 days post infection were fed on C57BL/6 mice and parasitaemia was monitored until day 14 post mosquito bite using Giemsa-stained blood smears.

### Recombinant PbP47 and antibody production

*PbP47* ^*opt*^ was engineered to contain codons allowing optimal expression in *E. coli*. A *PbP47* ^*opt*^ fragment lacking the signal peptide and the C-terminal hydrophobic domain was PCR-amplified with 5-GACAAGCTTGCGGCCGCAATTATATTCCCTAATGGATATGTC-3 and 5-TGCTCGAGTGCGGCCGCTTTGGAAGATGATATTTTTAATTCC-3 primers and infusion cloned into the *NotI* site of the pET-32b plasmid that carries N-terminal hexahistidine and thioredoxin tags (Novagen). Transfected shuffle T7 *E. coli* cells (NEB) were grown at 30 °C, induced with isopropyl-1-thio-β-d-galactopyranoside at 19 °C for 16 h, harvested by centrifugation, lysed using Bugbuster-lysonase (Novagen) and supplemented with a protease inhibitors cocktail (cOmplete EDTA-free, Roche). Cell debris was removed by centrifugation. The insoluble fusion protein was extracted from inclusion bodies using the inclusion body solubilization reagent (ThermoScientific), purified by cobalt affinity chromatography using TALON resins (Clonetech) under denaturing conditions in 8 M Urea in PBS, pH 7.4, and eluted using 250 mM imidazole in PBS pH 7.4. Protein refolding was achieved by stepwise decrease of Urea concentrations in PBS. Protein samples were analyzed by sodium dodecyl sulfate polyacrylamide gel electrophoresis (SDS-PAGE) and western blots to determine purity. Polyclonal antibodies were purified from pooled sera of two immunized rabbits (Eurogentec).

### Western blot and immunofluorescence assays

Parasite proteins were extracted with 1% Triton in PBS supplemented with protease inhibitors. Protein extracts were boiled under reducing conditions in Laemmli buffer, separated using 10% SDS-PAGE, transferred to a polyvinylidene difluoride (PVDF) membrane and detected using the rabbit α-PbP47 and a goat α-GFP (Rockland chemicals) antibodies at 1:100 and 1:1000 dilutions, respectively. Secondary horseradish peroxidase conjugated donkey α-goat IgG (Abcam) and goat α-rabbit IgG (Promega) antibodies were used at 1:5000 and 1:10000 dilutions, respectively. Gametocytes and ookinetes were fixed in 4% para-formaldehyde (PFA) in PBS for 20–25 min, smeared on glass slides and air-dried prior to blocking and antibody staining. Dissected midgut tissues were fixed after blood bolus removal in 4% PFA in PBS for 30 min, washed three times in PBS for 10 min, and blocked and permeabilized in 1% BSA and 0.2% Triton in PBS prior to antibody staining. Rabbit α-PbP47, rabbit α-TEP1^29^ and mouse α-P28 were used at 1:100, 1:300 and 1:1000 dilutions, respectively. Secondary Alexa Fluor 647 goat α-rabbit and 568 goat α-mouse IgG antibodies (Life technologies) were used at 1:1000 dilution. Images were acquired using a Leica SP5 MP confocal laser-scanning microscope, processed by deconvolution using the Huygens software and visualized with ImageJ.

### Gene silencing in *A. gambiae*

cDNA was prepared from total RNA extracted from adult *A. gambiae* and was used to produce *CTL4*, *LRIMI* and *TEPI* dsRNAs using primers reported previously^27, 36^. DsRNAs were generated with the T7 high yield transcription kit (Life technologies), purified using the RNeasy kit (Qiagen) and injected into the thorax of *A. gambiae* mosquitoes using the Nanoject II microinjector (Drummond Scientific) and glass capillary needles (0.2 μg in 69 nl per mosquito). Injected mosquitoes were allowed to recover for 2–3 days prior to infection with *P. berghei*.

### Macrogamete to ookinete conversion assays

For *in vitro* assays, 100 µl of a 24 h *in vitro* ookinete culture was pelleted, washed in PBS and resuspended in RPMI 1640. For *in vivo* assays, the blood bolus of 10 mosquitoes at 20–22 hours post blood feeding was pelleted, washed in PBS and resuspended in RPMI 1640. The suspension was incubated with a Cy3-labeled P28 antibody (1:50 dilution) for 10 min on ice. Conversion rates were calculated as the percentage of P28-positive ookinetes to P28-positive macrogametes and ookinetes.

### Genotypic analysis of transgenic parasites

Purified blood stage parasites were obtained after white blood cell removal using CF-11 columns (Whatman) and red blood cell lysis in 0.17 M ammonium chloride on ice for 20 min. Genomic DNA was extracted using the DNeasy kit (Qiagen). Detection of the wild type or knock out loci was achieved by PCR using primers as described^21^.

### Imaging and enumeration of parasites

Mosquito midguts were dissected and fixed in 4% (v/v) PFA in PBS for 20 min at room temperature, washed twice for 5 min in PBS and mounted on glass slides in Vectashield® (VectorLabs). Midgut and salivary gland sporozoite numbers from homogenates of 25–30 infected midguts or salivary glands were counted with a hemocytometer.

### Statistics

The Mann-Whitney *U*-test and generalized linear mixed models (GLMM) were used to determine statistical significance in parasite loads. GLMM analyses were performed in R (version 2.15.3) using the Wald Z-test on a zero-inflated negative binomial regression (glmmADMB). The various treatments were considered as covariates and the replicates as a random component. Fixed effect estimates are the regression coefficients. The infection prevalence and prevalence of melanized parasite presence were analyzed using the chi-square goodness of fit test. The unpaired Student’s *t*-test was used to determine statistical significance in TEP1 binding assays.

## Electronic supplementary material

Supplementary Info
