# Supplementary material for: Plasmodium berghei P47 is essential for ookinete protection from the Anopheles gambiae complement-like response
Source: Sci Rep. 2017 Jul 20;7:6026. doi: 10.1038/s41598-017-05917-6 (PMC5519742; doi:10.1038/s41598-017-05917-6)
Supplement: Supplementary file 1 — Supplementary Info [file 41598_2017_5917_MOESM1_ESM.pdf]

Plasmodium berghei P47 is essential for ookinete protection from the *Anopheles gambiae* complement-like response

Chiamaka Valerie Ukegbu, Maria Giorgalli, Hassan Yassine, Jose Luis Ramirez, Chrysanthi Taxiarchi, Carolina Barillas-Mury, George K. Christophides and Dina Vlachou

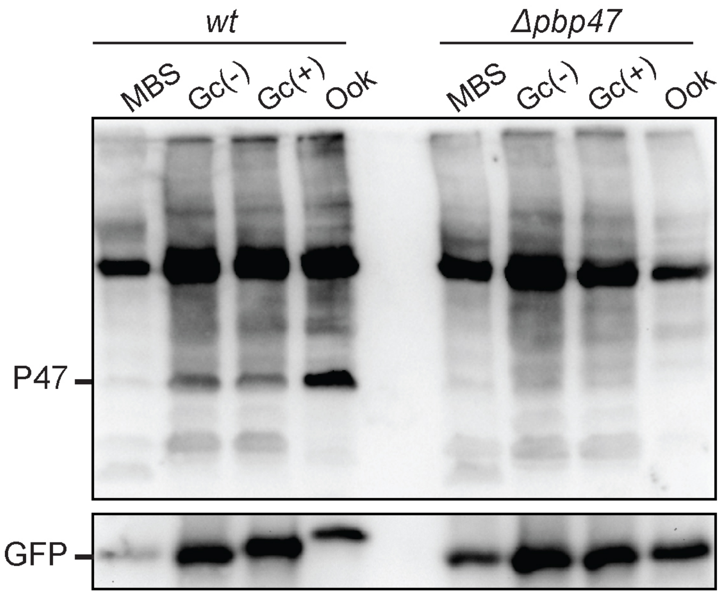

Supplementary Figure S1: Western blot analysis of protein extracts of *ANKA 507m6cl1* (*wt*) and *Δpbp47* parasites using the PbP47 and GFP (control) antibodies. In *wt* parasites, a highly specific band at the expected P47 molecular weight is detected in non-activated gametocytes (Gc(-)), activated gametocytes (Gc(+)) and *in vitro* produced ookinetes 24 hours post gametocyte activation (Ook). This band is absent from purified mixed blood stages (MBS) and all stages of *Δpbp47* parasites. A non-specific band of about 75 kDa is present in all stages of both parasite lines.

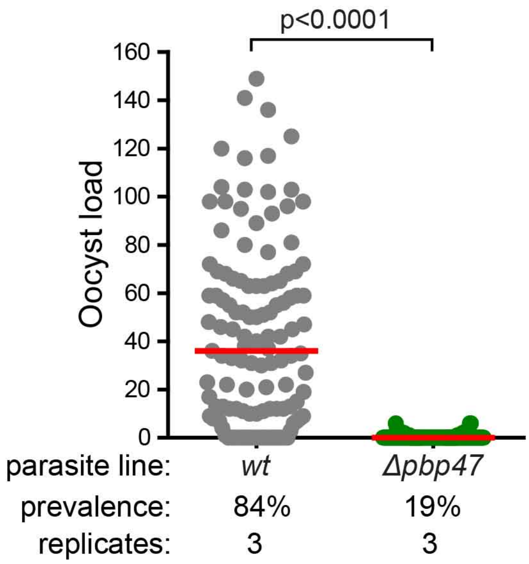

Supplementary Figure S2: Phenotypic analysis of *ANKA 507m6cl1* (*wt*) and *Δpbp47 P. berghei* infections of *A. gambiae* naïve mosquitoes. The median number of oocysts is shown with a red line. The infection prevalence (percentage of midguts harboring at least one oocyst) is shown. Statistical significance was determined with the Mann-Whitney *U*-test.

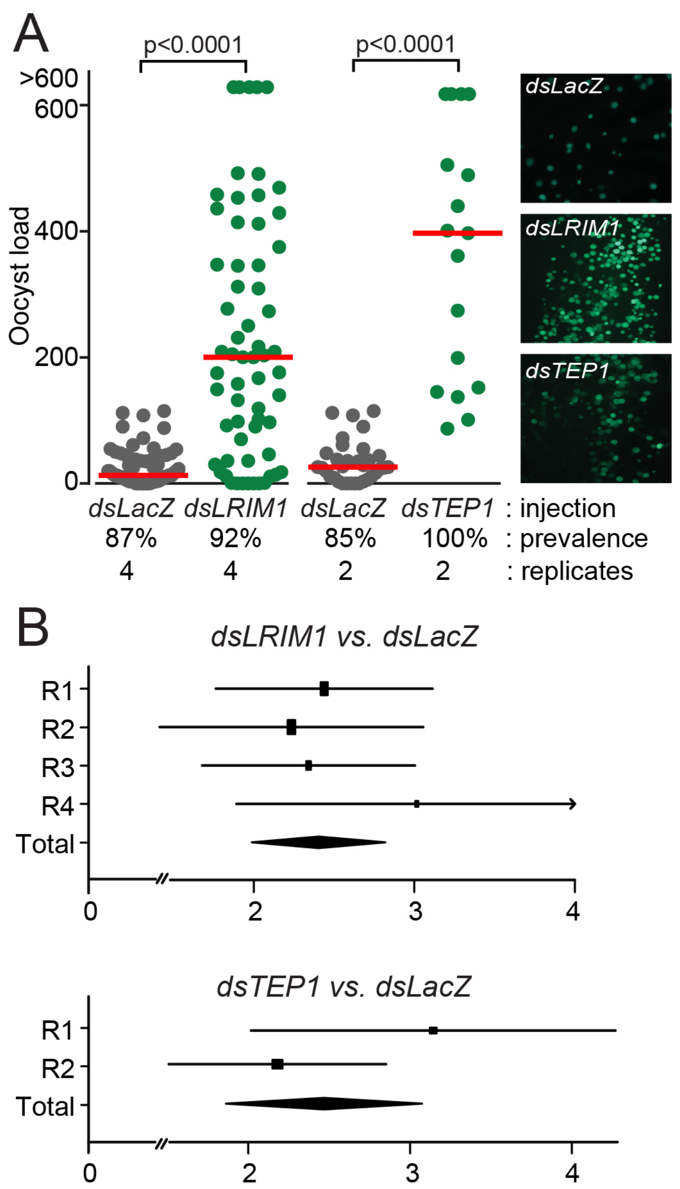

Supplementary Figure S3: Phenotypic analysis of *wt P. berghei* infections of *A. gambiae*. a, Effect of *dsLacZ* injections and *LRIM1* and *TEP1* silencing on *ANKA 507m6cl1* (*wt*) infection intensity and prevalence. The red line shows the median infection intensity (number of oocysts per midgut). The infection prevalence (percentage of midguts harboring at least one oocyst) is shown below the graph. Fluorescence images of *A. gambiae* midguts are representative of each set of experiments and were taken at X10 magnification. Statistical analysis of the oocyst loads was performed with a Mann-Whitney *U*-test. **b,** Forest plots of GLMM analyses of the infections shown in (**a**). The variation of the fixed effect estimate in each (squares) and all (diamonds) replicates (R) is shown (±95% confidence interval, glmmADMB). The square size is proportional to the sum of midguts analyzed in each replicate.

Table S1: Effect of *LRIM1* and *TEP1* KD on *Δpbp47* infection in *A. gambiae*

Parasite	DsRNA	No of replicates	No of midguts Total (replicates)	No of infected midguts Total (replicates)	Oocyst load Arithmetic mean	Median	Parasite range	*P* value
Δpbp47	*LacZ*	4	131 (26,30,36,39)	21 (7,8,4,2)	0.52	0	0-12	<0.0001
	LRIM1	4	91 (24,18,29,20)	86 (23,15,28,20)	49.5	22	0-505	
Δpbp47	*LacZ*	3	95 (36,39,20)	8 (4,2,2)	0.14	0	0-5	<0.0001
	TEP1	3	74 (35,21,18)	66 (32,20,14)	46.6	36.5	0-261	
wt	*LacZ*	4	82 (21,28,22,11)	71 (19,24,21,7)	23.7	13	0-115	<0.0001
	LRIM1	4	62 (24,21,8,9)	57 (23,17,8,9)	238.1	200	0-900	
wt	*LacZ*	2	33 (22,11)	28 (21,7)	33.7	26	0-115	<0.0001
	TEP1	2	17 (8,9)	100 (8,9)	397.5	397	87-910	

Δpbp47 and *wt* parasite infection of *LRIM1* or *TEP1* silenced *A. gambiae* mosquitoes was assessed 10 days post blood feeding. Statistical significance for oocyst load was determined using the Mann-Whitney U test.

Table S2: Effect of *TEP1* KD on *ΔPb48/45* oocyst numbers in *A. gambiae* midguts

Parasite line	dsRNA	Number of experiments	Number of midguts	Prevalence (%)	Infection intensity Arithmetic mean	Median	Parasite range
ΔPb48/45	*LacZ*	1	35	0	0	0	0
	TEP1		31	61.29	2.87	2	0-15
ΔPb48/45	*LacZ*	2	46	6.52	0.09	0	0-2
	TEP1		57	54.39	1.18	1	0-5

ΔPb48/45 parasite infections of *LacZ* KD, and *TEP1* KD *A. gambiae*. Prevalence illustrates the percentage of midguts with at least one oocyst. Parasite range shows the minimum and maximum number of oocysts detected.

Table S3: Midgut and salivary gland sporozoite numbers in *dsLacZ*-injected and *LRIM1* knockdown *A. gambiae*

		Midgut sporozoites		Salivary gland sporozoites		
Parasite	DsRNA	Mean	SE	Mean	SE	Mouse infectivity
Δpbp47	*LacZ*	42	29	0	0	0/6
Δpbp47	*LRIM1*	4,156	464	2,398	79	6/6
wt	*LacZ*	7,790	785	8,622	673	6/6
wt	*LRIM1*	17,343	1,988	16,060	665	6/6

The table reports *Δpbp47* or *507m6cl1* midgut (oocyst) and salivary gland sporozoite numbers from two biological replicates of *dsLacZ*-injected and *LRIM1* silenced *A. gambiae* infections. For each replicate, sporozoite numbers were determined from 25 homogenized midguts and salivary glands at days 15 and 21 post infection, respectively. Sporozoite infectivity was assessed after allowing infected mosquitoes at 21 days post infection to feed on C57/BL6 mice in bite-back experiments. Mouse parasitaemia was monitored for 14 days post mosquito bite. SE represents the error calculated from the average of the two biological replicates.

Table S4: Number of P28 positive *Δpbp47* and control *ANKA 507m6cl1* ookinetes in the midguts of *A. gambiae*

Infection experiment	Parasite line	No of midguts	P28+ ookinete range	Arithmetic mean	Median
1	*Δpbp47*	10	0-91	44	47
1	*wt*	5	15-284	151	124
2	*Δpbp47*	5	86-134	114	117
2	*wt*	8	0-323	220	270
3	*Δpbp47*	9	0-53	22	18
3	*wt*	13	5-446	130	78
4	*Δpbp47*	6	19-141	92	101
4	*wt*	8	244-701	458	406
5	*Δpbp47*	16	7-64	23	19
5	*wt*	18	0-752	233	139

P28-positive ookinetes in the mosquito midgut were enumerated at 24-26 hours post mosquito blood feeding using fluorescence microscopy.

Table S5: Melanized *ANKA 507m6cl1* (wt) and *Δpbp47* ookinetes in *CTL4* knockdown *A. gambiae*

Parasite	No of replicates	No of midguts	No of midguts with melanized ookinetes	Melanized ookinete load		Melanized ookinete range
		Total (replicate)	Total (replicates)	Arithmetic mean	Median	
wt	3	58 (32,12,14)	44 (24,9,11)	82.3	47	0-418
Δpbp47	3	100 (31,40,29)	77 (24,28,25)	44.3	13	0-286

The table shows the number of melanised *wt* and *Δpbp47* parasites in three independent biological replicates in the midguts of *CTL4* silenced *A. gambiae* 6 days post blood feeding.
